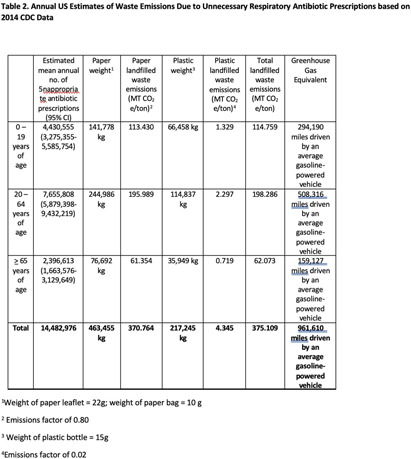# Greenhouse Gas Emissions Due to Unnecessary Antibiotic Prescriptions for Respiratory Diagnoses

**DOI:** 10.1017/ash.2024.166

**Published:** 2024-09-16

**Authors:** Emily Spivak, Adam Hersh, Jessica Tobin, Alexis Lee

**Affiliations:** University of Utah School of Medicine and Salt Lake City VA; University of Utah; University of Utah Health

## Abstract

**Background:** Healthcare accounts for 8.5% of total US greenhouse gas emissions (GHGE), with US healthcare the main contributor. Yet little effort has been made to measure healthcare related GHGE. Specifically, GHGE related to unnecessary antibiotic prescriptions is unclear, and to our knowledge, no one has used estimates of GHGE of unnecessary antibiotics as an antibiotic stewardship tool. We aimed to measure GHGE from solid waste associated with unnecessary antibiotic prescriptions for respiratory conditions. **Methods:** We calculated emissions for an outpatient prescription including the plastic bottle, paper leaflet, and paper bag (photos) based on the weight of each item multiplied by US Environmental Protection Agency (EPA) GHGE factors. Emission factors depend on waste type and treatment method which we assumed to be landfilled. To estimate unnecessary antibiotic prescriptions for respiratory infections, visits from nine University of Utah Health Urgent Care Centers from 2019-2022 were electronically identified and included if they had an ICD-10-CM code for a respiratory diagnosis where antibiotics are not indicated. Waste emissions of the paper and plastic in an individual prescription were then multiplied by the number of unnecessary respiratory antibiotic prescriptions for designated time periods to arrive at total landfilled waste emissions. We used similar methods applied to published 2014 data from CDC to estimate national waste emissions due to unnecessary antibiotic prescriptions for respiratory infections. Finally, we used the EPA’s GHG Equivalencies Calculator to convert emissions into tangible GHGE for providers and patients. **Results:** A prescription has 32g of paper and 15g of plastic waste. Among 124,461 urgent care visits (Table 1) in 2019-2022, 18,531 (14.9%) received an antibiotic. This equates to 593 kg of paper waste and 278 kg of plastic waste leading to a total landfilled waste emissions of 0.479 MT CO2e/ton. Using the EPA GHG Equivalencies Calculator, this equates to driving an average gasoline-powered car 1,228 miles. There were 14,482,976 unnecessary antibiotic prescriptions (Table 2) in the US for respiratory infections in 2014. Our estimates suggest these prescriptions led to 375.109 CO2e/ton of GHGE, the same as driving 961,610 miles by an average gasoline-powered vehicle. **Conclusion:** Unnecessary antibiotic prescriptions are associated with substantial GHGE. This estimate demands further evaluation across diagnoses and care delivery sites, and most importantly action. Additionally, the large GHG contribution of unnecessary antibiotics should be used as a stewardship tool to highlight low-value care that is likely contributing to global climate change.

**Figure:**
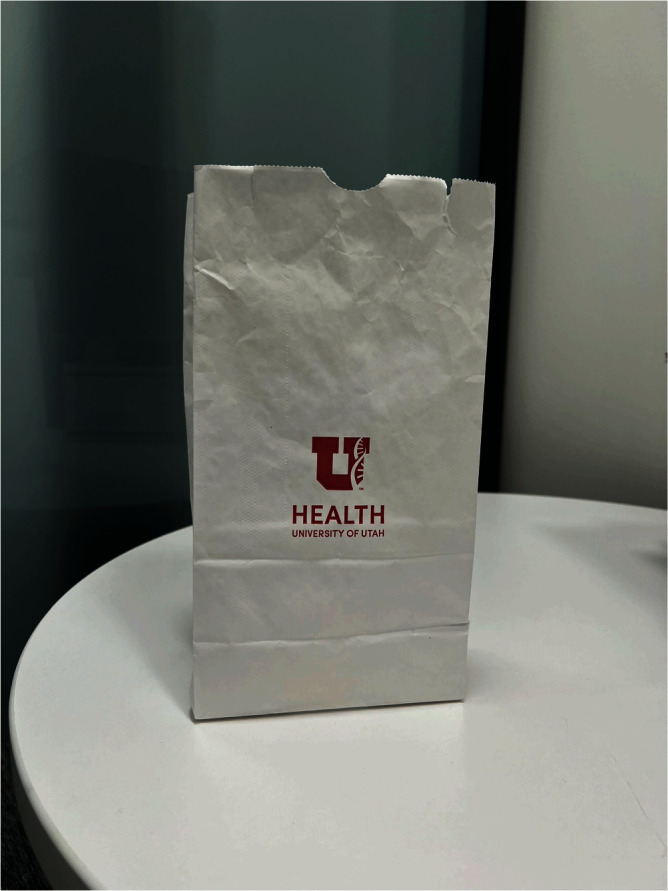

**Figure:**
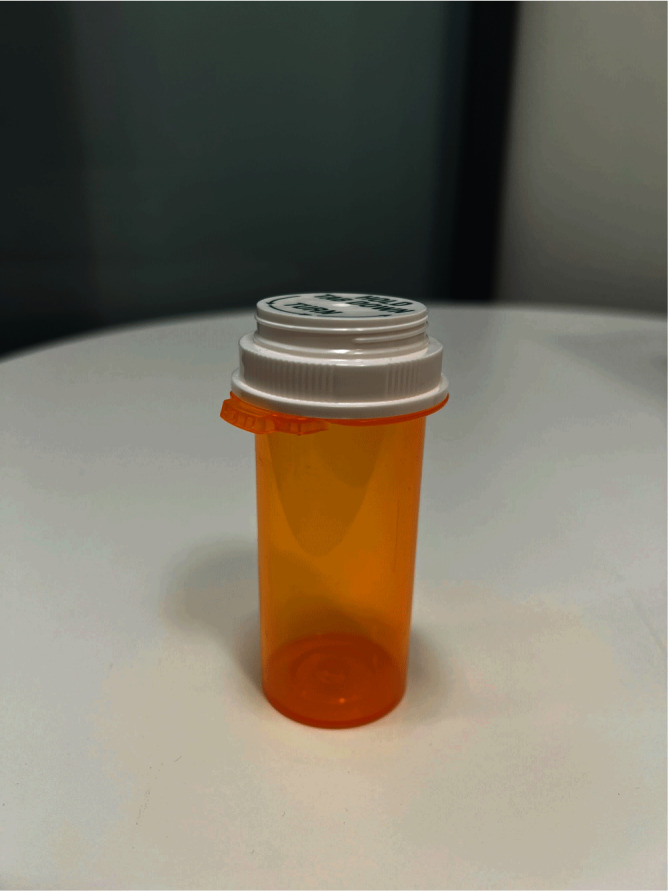

**Figure:**
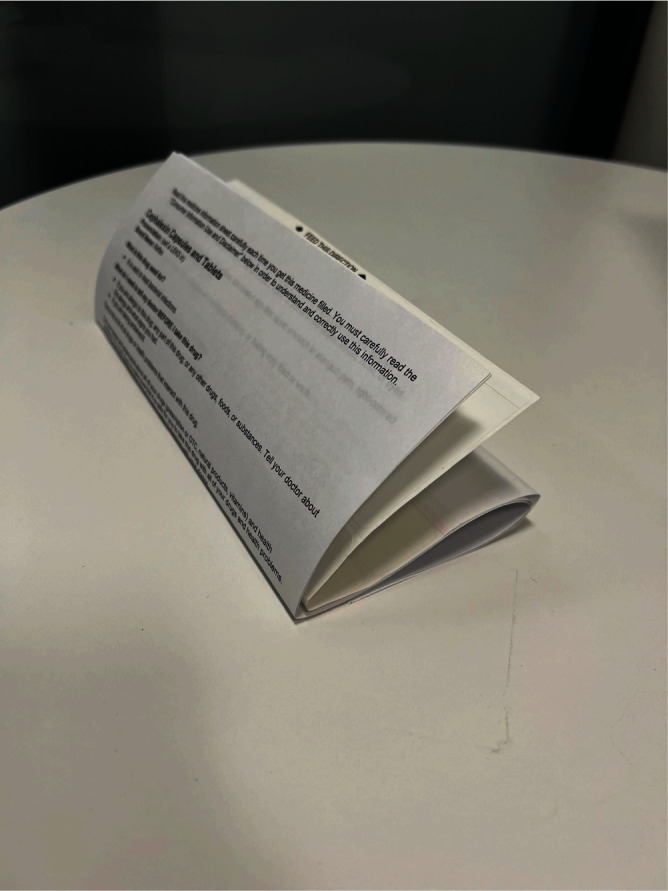

**Figure:**
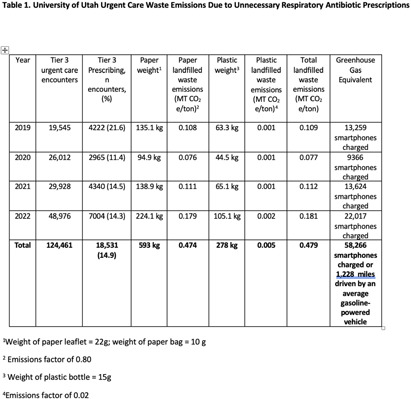

**Figure:**